# Annual Address of George Wigg, M. D.

**Published:** 1890-08

**Authors:** 


### ANNUAL ADDRESS OF GEO. WIGG, M. D.,

**President of the Homœopathic Medical Society of the State of Oregon, delivered at its Fourteenth Annual Session, held at Portland, Oregon, Tuesday, May 13th, 1890.**

Ladies and Gentlemen :—Time, ever swiftly rolling onward with its burden of care, as well as of joys and pleasures, has again favored us with another opportunity of meeting together to exchange fraternal greeting, and review our labors of the past twelve months.

With some of us these golden moments will pass—are passing rapidly away with each changing year ; such being the case, let us make the most of our time in giving and receiving all the good in our power.

We are assembled at this time under circumstances of no ordinary character—peculiarly blessed at the hands of the Great Physician, in the enjoyment of full health and strength, and of our reasoning faculties, blessed in our basket and in our store, in our going out and in our coming in. Let us then with thankful hearts look up to the Giver of all good, and invoke His sanction and blessing upon all we may say or do at this meeting; may He who is the fountain head of all wisdom, expand our minds, enlighten our understandings, so that we may the better grasp the great problem of restoring the sick to health.

Since our last meeting, two of our number have fallen from the ranks of Homœopathy in this State, Dr. Pohl, late of Portland, and Dr. Resdon, late of Salem. At an hour they were not aware of, their spirits took their departure from the tenements of clay. We shall not see their faces nor hear their voices in our midst again ; but in the deathless hereafter, our spirits, with theirs and Hahnemann's, Dunham's, Hering's, and a host of others who have departed from the battle-field of life, shall together walk the palace of the skies. For we know there is something that lives, the dust can but cover its dust.

In taking a retrospective view of the past twelve months, we stand awe-stricken as the panorama passes before the mind's eye. City after city has been laid low by the devouring element, fire. Thousands of human lives have been sacrificed in that ever-restless element, water, while catastrophes from other causes have been legion. Yet, amidst it all, the physician has calmly performed his mission of toil and mercy, and Homœopathy has brought forth golden blocks for the Temple of the Art of Healing.

For fourteen years this Society has annually congregated, and the results of these gatherings are manifest in all parts of Oregon. Already there are thousands of persons living in Oregon who can testify to the superiority of Homœopathy over every other known system of medicine. This fact ought to stimulate us to still greater effort in behalf of those grand truths which it teaches.

I repeat, "those grand truths." For when Samuel Hahnemann, in the year 1796, announced to the world the law of *similia*, he proclaimed a truth high as heaven, deep as hell, and lasting as eternity; and because of this truth, he was hated by physician and apothecary alike, and in some kingdoms his practice was interdicted by law, and the law went so far as to forbid him from dispensing his own remedies.

How much better it would have been for humanity at large, had the old-school doctors assisted Hahnemann in his search for proof of the law he taught. Hahnemann threw down the gauntlet when he said: "Either prove or disprove the truth of what I say—if true, acknowledge it; if false, publish it to the world." But instead of doing so, they heaped upon that head, which was the peer of them all, calumny, and not content with that, they drove him from the land of his birth, causing him to become a stranger in a strange land. No doubt he thought, as he turned his back upon his native land, Was ever grief like unto mine. But knowing the truth of what he taught, and seeing, away in the far distant gloom, faint rays of that sun which would arise and throw its illuminating power into the most secret recess of allopathic ignorance and deceit, he, like a well-inflated balloon in a calm atmosphere, moved smoothly through it all, landing at last triumphant, and to-day he, though dead, lives as "Hahnemann the Conqueror."

His enemies might down him, stone him, yea! have slain him, his blood would only have given nourishment to his convictions and the truth he had already proclaimed would have still grown and reflected back upon their guilty conscience—the law, "*Similia similibus curantur*," which even now haunts and troubles them.

In the year 1834 Homœopathy was represented by an old-school doctor as being nothing but a frightful abortion, with a big body, goat hoofs, crooked arms and long fingers, fox's eyes, donkey's ears and a hydrocephalic head, and like every other work of deceit and darkness, must of itself fall to the ground.

"And 'twas thus they shouted and they spouted;
Then they spouted and they shouted;
But the people jeered and blouted,
At their damned infernal lies."

Had it been in the power of the allopaths to have done so, they would have burned Hahnemann and his adherents at the stake, even to-day are they not plotting the destruction of the name Homœopathy—and no stone will they leave unturned that will help them in their work of persecution. But Homœopathy is powerful, and, being founded upon truth, cannot be destroyed.

In an article published in the *Medical World* of January, 1890, Vol. VIII, page 24, the editor, in speaking of the homœopathic law of cure, says: "A prudent man will not fasten himself to any so-called law of cure, because no such law exists." Great God! what an expression. "No law." A world of men, women, and children, bowing beneath the curse of disease, and an army of noble-minded physicians toiling incessantly to find the best ways and means by which disease may be cured, life prolonged, and sorrowful hearts made happy and "no law." Go! stand at the base of yon snow-capped mountain—watch those little streamlets creeping from their white bed, see them form into brooks, then watch the brooks as they form into creeks, the creeks into rivers, and the rivers into the grand old ocean, there forming a highway for the commerce of the whole world, and "no law." Again, watch the sun as it rises on a bright July morning, gilding the rosy-fingered morn with its crimson; or the moon, as it climbs from behind yon hill, veiled in the silvery mist of night; or, if you please, you may watch the Great Bear as he swings in his circle, around the polar star, and "no law." Then go and stand by the bed of those suffering from disease and tell me if there is "no law," why in typhoid fever one tongue is dry and brown, with a red streak in the middle, and another red, dry, cracked and stiff; or why one is white and another yellow? Why is it in scarlet fever, one's tongue is of a deep red color, covered all over with white blisters, and another's white with red edges?

If there is "no law," how comes it that in rheumatism, one's pains are aggravated by cold, and better by warmth; and another's is made worse by heat, and cease altogether by the application of cold; or why are one's pains aggravated by motion, and another's by rest?

Ladies and gentlemen, there is a law, and you know it; and this law of cure is as universal as the universe. How a physician, ignorant of this law, must grope around the sick form of his patient, "as gropeth the blind, and know not at what they stumble!"

Remember, that ignorance of how to apply this law in disease does not destroy it. See yon powerful engine, that by a well-known law can be made to attain a speed of one hundred miles per hour; yet ignorance of the law of evaporation and condensation, on the part of the engineer, may cause its boiler to explode and scatter death and destruction all around it, but the bursting of the boiler would not destroy the law that should have governed it. So with the homœopathic law of cure, it will do for the sick what no other known law can accomplish; yet, for lack of knowledge in its application, death may result.

In Monday's issue of *The Oregonian*, January 20th, is an article from the pen of Dr. Van Dike, of Grant's Pass, Oregon, in which he states that "there are no allopathic doctors, allopathic teachers, and never have been; we simply call ourselves regular practitioners." But the Doctor does not inform us in what he or his school are regular, nor upon what grounds they have arrogated unto themselves such a ridiculous claim. Further on he informs us "that the falsely called allopaths have made all the great improvements and discoveries in medicine, surgery, and the allied sciences, and McDowell, Sims, Biglow, Sager, Gross, and the other illustrious men in medicine and surgery belonged to the so-called allopathic school. Show me such an array of names in any other school, and I will show you a few white blackbirds."

We know the above-named gentlemen to be men of eminence among their professional brethren, and we know further that Samuel Hahnemann, the founder of the homœopathic school of medicine, was the peer of them all; and it was Hahnemann, and such men as Hufeland, Guernsey, Lippe, Bœnninghausen, Wesselhœft, and a host of others, not members of the allopathic school, that have done, and are still doing more to bring order out of medical chaos than any other men in this or any other age. These men brought the truths of Homœopathy to bear with such force against the citadel of the old school of medicine as to cause its ponderous machine to move forward, and to-day one can hear the grating of its bearings, as friction takes off the rust and dust of ages.

The influence of homœopathic teachings and practice on the allopathic school of medicine has been such as to constrain them to relinquish those barbarous methods which were in vogue before the days of Hahnemann. Had Homœopathy accomplished nothing more than this, and could it claim no more favorable results in the treatment of the sick than the allopaths, even then it still ranks pre-eminent in medicine.

At the fifteenth session of the State Legislature an Act was passed regulating the practice of medicine and surgery in this State. This Act gave the Governor power to appoint three persons from among the most competent physicians living in

Oregon. The Governor, for some reason known only to himself, appointed three allopathic physicians, and in so doing has given to the State a one-legged Medical Board of Examiners, with power to issue certificates to any person or persons who in their judgment are qualified to practice medicine and surgery.

Now we would like to know by what means this one-legged Board is going to test the qualifications of homœopathic physicians, seeing the Board itself is as ignorant of the law of the homœopathic art of healing as a sturgeon is of the ten commandments, for Homœopathy is not taught in their colleges, neither will they allow their students to investigate it.

Hence I say that the three gentlemen composing the Medical Examining Board of the State of Oregon, being ignorant of the *whole* science and art of medicine, are not qualified to judge of the ability of those doctors who have graduated from homœopathic colleges to practice as homœopathic physicians. And, further, seeing that the allopaths are opposed to Homœopathy, and hold it up to the world as nothing but a humbug, how can they conscientiously recommend a homœopath to the people of this or any other State?

And, again, let us suppose that the Governor had seen fit to appoint thee homœopaths as medical examiners, how could they, not believing in the allopathic system of practice, recommend the students of their colleges as qualified to practice medicine? Suppose that at the next meeting of the Legislature an Act to regulate the practice of preaching in the State of Oregon should be passed and the Act give to the Governor power to appoint three clergymen from among the clergy living in this State, and suppose the Governor appointed three priests, members of the Roman Catholic Church, who should constitute a Board of Preacher Examiners, with power to grant or withhold a license, just as they saw fit, do you think the clergymen of other denominations would for one moment tolerate or submit to such a law? Verily, no. They would demand that each Church regulate its own household.

We say, give us an Examining Board composed of nine members, three from each of the leading schools of medicine; and if you will not do that, then we demand in the name of freedom and righteousness, separate Examining and Licensing Boards for each of the legally organized medical schools of this State.

Need we remind our opponents that the Persian, in his day, had the richest, vastest empire in the world? He boasted that his laws were unalterable, and his wisdom so great that his opinions were never changed. A small but sturdy people arose on his borders—the Greek, subtle, supple, and great students of nature. With tents that covered the land, and sails that whitened the sea, the Persians came on to destroy their foe. The result was Salamus and Marathon.

In due time the Greek arose in his turn, over-ran the empire of his enemy without sheathing his sword, trampled the unalterable laws in the dust, and divided out all his provinces among the followers of his camp.

You must remember that it is no longer Samuel Hahnemann, fleeing before the wrath of his enemies, oh! no. Homœopathy turned its face to the face of its adversary long ago, and being urged on, will fight till she conquers. Like the everlasting Gospel, its power and influence are acknowledged in every corner of the world. Never in its history has Homœopathy stood so high as it stands to-day; never before did the rich and influential give of their substance for the spread of its cause as they are giving now. It is here a college, there a hospital, and on this street an ambulance, and on that a free dispensary, with a large staff of physicians at work by day and by night.

Time would fail me to tell how it is spreading all over the world. In America we have nine thousand five hundred practitioners, fifty-seven hospitals, several State-supported lunatic asylums, one hundred and ten societies, twenty-six periodicals, as well as a number of annual publications containing reports of transactions.

The British province of Canada contains a large number of homœopathic practitioners, whilst in Mexico, Germany, Austria, Hungary, Switzerland, France, South America, Australia, England, China, New Zealand, Hindoostan, and other places the growth of Homœopathy in the past few years has been marvelous. Gradually, at first unrecognized, surrounded by all manner of weeds, it has at last grown into an oak of God, and suffering mankind are now healed under its beneficial shade.

And now, my dear fellows, I hope that after the business here has been transacted we will each return to our several posts of duty, determined to discharge faithfully the duty we owe to ourselves and to our patrons. Let us strive to do all in our power to cultivate a more fraternal feeling with our professional brethren everywhere, and should our lives be spared, may we return to our next annual meeting, laden with the garnered experience of another year, to give as a free contribution to the cause of Homœopathy.

Hoping that your deliberations may be harmonious, and your intercourse with each other, while here, fraternal—I will now thank you all for the courtesies you have manifested toward me during the two years I have presided over this society, and I shall ever remember those years as ones of pleasure, and pride myself in the fact that, as President of the Homœopathic Medical Society of the State of Oregon, I presided over a society whose profession is inferior to none, and whose art the noblest that ever taxed the intellect of man.

